# Determinants of proliferation and involution in infantile hemangioma: macrophage crosstalk, endothelial plasticity, and metabolic rewiring

**DOI:** 10.3389/fped.2026.1847957

**Published:** 2026-07-22

**Authors:** Beichen Cai, Qian Lin, Ruonan Ke, Lu Chen, Xiaofen Wan, Xuejun Ni, Xiuying Shan, Biao Wang

**Affiliations:** 1 Department of Plastic Surgery, The First Affiliated Hospital of Fujian Medical University, Fuzhou, China; 2 Department of Plastic Surgery, National Regional Medical Center, Binhai Campus of the First Affiliated Hospital of Fujian Medical University, Fuzhou, China

**Keywords:** endothelial-to-mesenchymal transition, ferroptosis, glycolysis, hemangioma stem cells, infantile hemangioma, macrophage polarization, propranolol

## Abstract

Infantile hemangioma (IH) follows a characteristic clinical course, yet the mechanisms that sustain rapid postnatal growth and later permit fibrofatty regression remain incompletely resolved. This gap has practical consequences, because some lesions threaten function, some respond incompletely to propranolol, and residual tissue change can remain important after growth arrest. Recent studies have expanded the field beyond classical angiogenic signaling to include phase-specific cell states, macrophage-endothelial crosstalk, and endothelial metabolism, but these advances are usually discussed separately. In this review, we prioritize human lesion tissue, patient-linked phase-resolved data, and primary hemangioma endothelial cell (HemEC)/hemangioma stem cell (HemSC) studies, while using cell-line and xenograft systems mainly for mechanistic dissection. Across these evidence tiers, the evidence points to a phase-linked model. Proliferative IH is supported by reciprocal signaling among hemangioma stem cells, endothelial cells, mural cells, and activated macrophages; enhanced glycolysis and selective amino acid use sustain endothelial growth; M2-skewed macrophage signals promote endothelial proliferation and differentiation; and HemSC-derived cues protect macrophages from ferroptosis through NRF2-GPX4. Involution appears to involve loss of this supportive niche, increased lipid peroxidation, and cell-state shifts that permit mesenchymal and adipogenic differentiation. However, endothelial-to-mesenchymal transition should not be treated as uniformly regressive: inflammatory EndMT linked to M1 cytokines differs from TGF-β1-driven programs that can still enhance migration and angiogenesis. That distinction is relevant for biomarker design and therapeutic targeting. We argue that the most informative next steps are phase-resolved human cohorts, spatial validation of macrophage and endothelial states, and early translational studies that pair standard therapy with rational inhibitors of glycolysis, PI3K/AKT/mTOR signaling, or macrophage survival pathways.

## 1 Introduction

Infantile hemangioma is the most common vascular tumor of infancy and is defined less by static histology than by a distinctive clinical trajectory. Most lesions appear within the first weeks of life, enlarge rapidly during early infancy, and then gradually regress over several years into a fibrofatty residuum of variable cosmetic and functional consequence. This natural history has clinical importance because the subset of lesions located near the eye, airway, liver, lip, or nasal tip can threaten vision, breathing, feeding, or long-term facial development. The 2019 American Academy of Pediatrics Clinical Practice Guideline formalized risk stratification and the need for timely referral of high-risk lesions, and the randomized trial by Leaute-Labreze and colleagues established oral propranolol as the therapeutic standard for problematic proliferative IH. Even so, the drug did not eliminate the underlying biological uncertainty. Not all proliferative lesions respond at the same rate, rebound after treatment withdrawal remains clinically relevant, and residual contour change, telangiectasia, or fibrofatty tissue may remain even when the vascular component regresses (1–3). The central clinical problem is therefore twofold: how the lesion is maintained in a proliferative state, and what drives the transition toward involution.

The traditional explanation for IH pathobiology has centered on dysregulated angiogenic signaling, relative hypoxia, and altered stem-cell behavior. These mechanisms remain important, but they do not fully explain several observations that are now hard to ignore. First, proliferative and involuting lesions differ in vessel density, immune-cell composition, oxidative stress, and differentiation state. Second, recent work has shown that macrophages in IH are not passive bystanders; depending on context, they can support endothelial expansion, suppress adipogenesis, or promote regression-associated endothelial plasticity. Third, endothelial and stem-cell metabolism is increasingly implicated in lesion maintenance, with convergent evidence for enhanced glycolysis and altered amino acid handling. These findings broaden the disease model from a narrow angiogenesis problem to a multicellular, phase-dependent process in which cell fate, metabolism, and the immune microenvironment are tightly linked. Yet the literature remains fragmented. Macrophage studies, metabolic studies, and cell-fate studies are often interpreted separately, and field-wide shorthand such as “M1 vs. M2” or “EndMT equals regression” can obscure meaningful biological heterogeneity.

This review addresses that gap by integrating the current evidence into a phase-specific framework of IH progression and involution. We prioritize human lesion tissue, patient-derived HemSC and HemEC models, and studies that explicitly compare proliferative and involuting disease. Animal models, immortalized cell systems, and THP-1 macrophage studies are treated as tools for pathway dissection rather than proof of human disease primacy. Our central thesis is that proliferative IH is maintained by a supportive niche composed of HemSCs, endothelial cells, mural cells, and activated macrophages, and that involution reflects the gradual breakdown or reprogramming of this niche rather than a single linear trigger. We further argue that three processes deserve to be interpreted together: macrophage-state transitions, endothelial metabolic rewiring, and cell-state plasticity leading toward mesenchymal and adipogenic outcomes. By separating established findings from plausible working models, we aim to clarify what is already defensible, what remains uncertain, and where the most tractable translational opportunities are likely to emerge.

The manuscript is organized accordingly. Section 2 defines the phase-specific cellular and evidentiary framework on which later interpretation depends. Section 3 focuses on processes that maintain the proliferative lesion, separating niche support, endothelial metabolic dependence, and macrophage survival mechanisms. Section 4 then addresses processes linked to exit from the proliferative state, with specific attention to endothelial plasticity, adipogenic remodeling, and the limits of a simple M1-to-M2 narrative. Section 5 translates this framework to biomarkers, therapeutic vulnerabilities, and early-phase trial logic, and Section 6 defines the main evidence gaps that should shape the next generation of human studies.

## 2 Cellular and phase-specific framework of infantile hemangioma

### 2.1 Natural history, lesion compartments, and why phase assignment matters

IH is often described in clinical terms as moving from proliferation to involution, but the biological meaning of those phases is more complex than a simple change in lesion size. Proliferative lesions contain dense vascular lobules, abundant endothelial cells, immature mural support, and a stromal compartment that includes stem-like cells, fibroblasts, and immune infiltrates. Involuting lesions show reduced endothelial cellularity, vessel maturation or collapse, increased adipogenesis, and progressive remodeling of the extracellular matrix. The disease therefore cannot be understood by examining endothelial proliferation alone. Each phase reflects a changing balance between endothelial growth, stem-cell differentiation, inflammatory support, and tissue remodeling.

**Figure 1:**
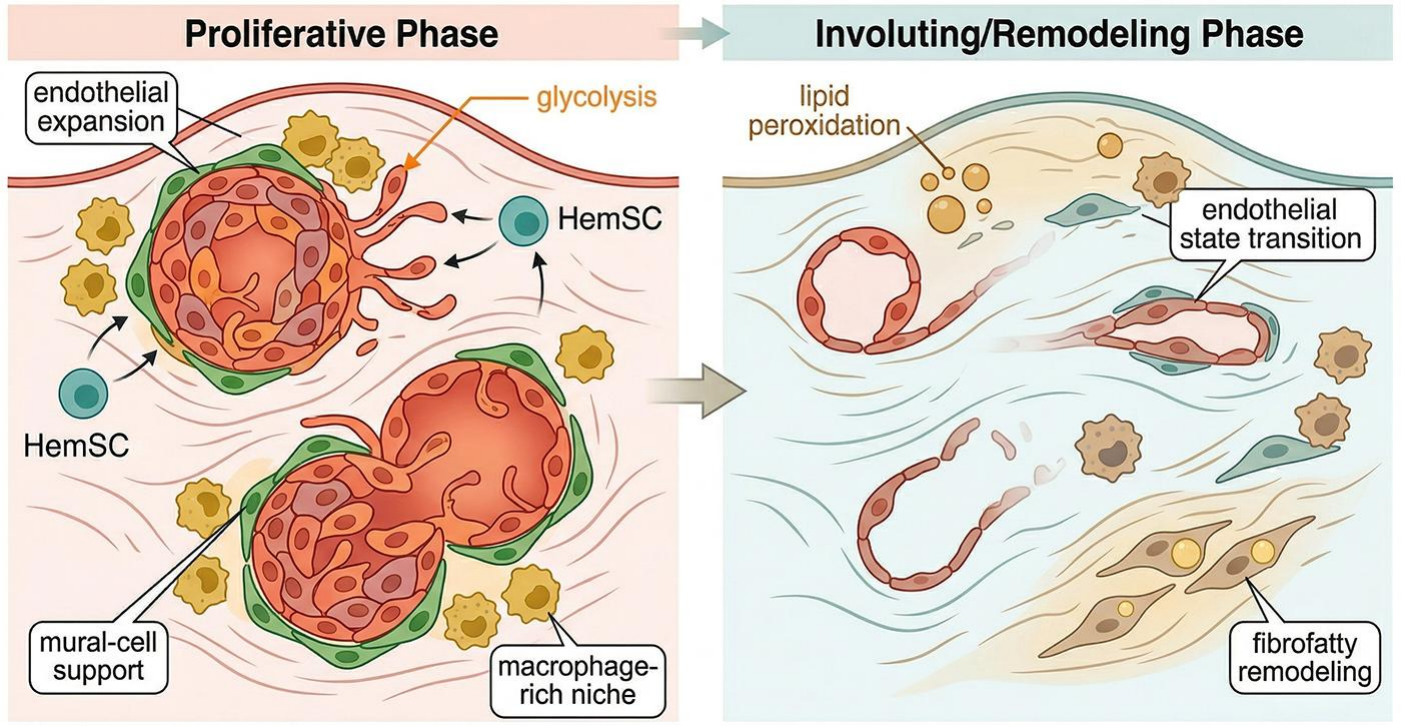
Temporal phase map of infantile hemangioma progression and involution. The left side summarizes the proliferative lesion as a cooperative niche in which HemSCs, heterogeneous endothelial cells, mural/perivascular cells, and macrophages support dense vascular growth, glycolysis, and angiogenic signaling. The right side summarizes the involuting/remodeling phase, with reduced vascular density, lipid peroxidation, endothelial state transition, adipogenic differentiation, and fibrofatty remodeling. The figure is a schematic synthesis of published evidence and does not present primary experimental data.

Recent single-cell work reinforces this point. In a 2025 study of 36,237 cells from three proliferative IH samples, endothelial cells displayed marked heterogeneity rather than a single uniform phenotype, and hemangioma mural cells accounted for more than half of the lesion. Ligand-receptor analysis suggested that mural cells release VEGFA and PGF, which can reinforce endothelial proliferation and angiogenesis. This atlas is important because it confirms that the proliferative lesion is not endothelium in isolation. It also fits well with earlier primary-cell studies showing that CD146-positive mural cells and PDGFR-β-positive perivascular cells from IH retain both proangiogenic and adipogenic potential (4–6). At the same time, the atlas is limited to proliferative tissue and does not yet define how these cell populations evolve during involution or in response to therapy. Even so, it provides a useful structural starting point for interpreting older pathway-focused work and suggests that any model of IH biology that excludes stromal and mural compartments is incomplete from the outset (Figure 1).

Phase assignment matters because the same pathway may have different implications depending on when and where it is measured. A marker associated with proliferative endothelial behavior in one setting may indicate regression-associated plasticity in another. This is especially relevant for macrophage polarization, oxidative stress, and EndMT, all of which are highly context dependent. For example, increased mesenchymal marker expression can mark endothelial destabilization that still supports invasion and angiogenesis, or it can represent a more terminal departure from endothelial identity that permits adipogenic conversion. Likewise, macrophage-associated cytokines can either support proliferation or drive regression depending on the cytokine mixture, cell source, and timing. A clinically useful framework therefore requires phase-resolved interpretation rather than pathway cataloging alone.

### 2.2 Human tissue evidence for phase-linked cellular states

The strongest anchors for disease relevance remain human lesion studies that compare proliferative and involuting IH. Several such studies support the view that phase transition in IH is accompanied by coordinated shifts in immune composition, oxidative stress, and differentiation state. Older histologic work established the clinical diagnostic value of GLUT1 and documented the mixed cellular composition of proliferative lesions. More recent studies have extended this to phase-linked immune and metabolic features. Zhang and colleagues showed that macrophage infiltration is more prominent in proliferative than involuting IH and that macrophage-associated cytokines are enriched in the proliferative phase. Wu and colleagues later demonstrated that involuting lesions are characterized by perivascular M1 macrophage infiltration together with endothelial Snail/Slug expression, consistent with inflammatory induction of EndMT. A 2020 tissue-based study further showed that proliferating IH specimens and primary cell lines express EndMT-associated markers, which supports the existence of endothelial state plasticity while leaving open the question of whether those changes are proangiogenic, regressive, or both depending on context (7–9).

Newer patient-linked data refine the metabolic dimension of this transition. Liu and colleagues compared nine proliferative and 12 involuting IH samples and found that 4-hydroxynonenal, a marker of lipid peroxidation linked to ferroptotic injury, is higher in involuting lesions, whereas GPX4 expression is higher in proliferative lesions. Importantly, GPX4 colocalized mainly with CD68-positive macrophages rather than endothelial cells, suggesting that ferroptotic resistance in the proliferative phase is concentrated in macrophages. This observation is clinically relevant because it shifts part of the survival program away from the endothelial compartment and toward the supporting immune niche. In parallel, KLF2 has emerged as a cell-fate-associated marker of lesion state: it is enriched in endothelial cells within proliferative IH and reduced in adipocytes during involution. Although KLF2 is not yet a clinical biomarker, these human-tissue observations support the broader principle that phase transition in IH involves linked changes in vascular identity, stromal differentiation, and immune support rather than a passive decline in angiogenesis alone (10, 11).

The evidence for phase-linked endothelial plasticity is less linear. The 2017 M1 macrophage study linked involuting lesions to M1 infiltration and regression-associated EndMT markers, whereas a 2025 TGF-β1 study found that HemECs can undergo mesenchymal marker induction while still displaying increased migration, invasion, and angiogenesis. Human multiplex immunofluorescence in that later study showed progressive changes in endothelial and mesenchymal marker colocalization across disease stages, but the functional interpretation of these marker shifts is not identical across contexts. The key implication is that human tissue supports the existence of endothelial state transitions in IH, but not all such transitions should be assumed to represent the same biological endpoint. When tissue markers are interpreted without regard to inflammatory context, exposure duration, or lesion phase, overstatement becomes likely (Table 1).

**Table 1:** Evidence hierarchy for mechanistic studies in infantile hemangioma.

Evidence tier	Typical material in the IH field	Most defensible conclusions	Main limitations for this review question	Representative references
Tier 1: Human lesion tissue and patient-linked biospecimens	Phase-resolved surgical tissue, immunohistochemistry, immunofluorescence, multiplex staining, phase-linked biospecimen comparison, single-cell atlases derived from human lesions	Disease relevance, phase association, lesion-compartment localization, coexistence of cell states within lesions	Mostly cross-sectional; small sample size; treated and untreated lesions are often mixed; cannot by itself prove temporal sequence or causality	(1, 4, 7, 8, 10)
Tier 2: Primary patient-derived cells and higher-fidelity functional systems	Primary HemSCs, primary HemECs, primary mural/perivascular cells, adipogenic and endothelial differentiation assays, phase-matched explant-like systems	Causal plausibility for cell-fate effects, endothelial plasticity, metabolic dependence, and stromal-endothelial signaling	Culture conditions may favor selected states; short-term assays can overemphasize motility or viability endpoints; still do not capture whole-lesion developmental timing	(5–7, 11, 13, 20)
Tier 3: Lower-fidelity mechanistic support systems	THP-1-derived macrophages, HUVEC comparators, immortalized endothelial models, xenografts and Matrigel implants, drug-screen platforms	Pathway dissection, rescue experiments, testing whether a candidate mechanism can alter lesion-like behavior	Cell identity drift, incomplete immune context, species differences, endpoint mismatch with human clinical outcomes, risk of overranking single-gene axes	(10, 12, 29)

Overall, the most established human findings are phase-linked differences in lesion cellularity, macrophage distribution, and oxidative-stress markers. By contrast, the precise ordering of endothelial plasticity, adipogenic remodeling, and macrophage-state change remains a synthesis built from cross-sectional tissue plus experimental studies rather than a directly observed sequence in patients.

### 2.3 What current model systems can and cannot tell us

The IH field depends on a mixture of patient tissue, primary cell cultures, xenografts, immortalized endothelial models, and surrogate systems such as THP-1-derived macrophages or HUVECs. Each contributes differently to causal inference. Human lesion tissue provides disease relevance and phase information but is usually cross-sectional and limited by small sample numbers, especially for involuting lesions and serial sampling. Primary HemSCs and HemECs offer better fidelity than generic endothelial lines and have been essential for showing effects on endothelial differentiation, adipogenesis, and angiogenesis. Xenograft and Matrigel implantation models allow testing of vessel formation and growth but do not fully capture the developmental timing, immune composition, or spontaneous involution seen in infants.

These limitations are especially important when interpreting macrophage and metabolic studies. The M2 exosomal MIR4435-2HG study used THP-1-derived M2 macrophages, HemEC assays, human tissue staining, and a nude mouse xenograft. This combination is strong for demonstrating a plausible signaling axis, but it does not prove that the same exosomal circuit is quantitatively dominant in untreated human lesions across time. Likewise, the macrophage ferroptosis study combined human lesion comparisons with THP-1 macrophages exposed to HemSC-conditioned medium and a mouse implantation model. This supports a role for GPX4-dependent macrophage survival, but it does not yet define which macrophage subsets in infants are responsible or whether similar biology holds under propranolol treatment. The same caution applies to many metabolic papers, in which upstream regulators are often defined by knockdown and rescue in cultured HemECs or XPTS-1 cells and then extended to xenograft growth. A related problem is treatment exposure: because propranolol and other interventions can themselves alter endothelial survival, differentiation, and cytokine programs, treated and untreated lesions should not be pooled uncritically when phase-specific mechanisms are being inferred (2, 10, 21, 22, 25).

At the same time, these models remain useful when interpreted with discipline. The most convincing mechanistic signals are those that recur across more than one evidence level. Increased glycolytic dependence is supported by differential expression in proliferative tissue, metabolic readouts in HemECs, and *in vivo* suppression after pathway inhibition. Macrophage involvement is supported by histology, cytokine profiling, cell-culture effects on HemSC behavior, and animal-model effects on vessel density. Endothelial plasticity is supported by patient histology, cytokine-treated HemEC assays, and adipogenic conversion studies. The field now needs models that preserve these strengths while reducing distortion. Co-culture systems that incorporate HemSCs, HemECs, mural cells, and primary human macrophages, together with spatially resolved human tissue studies, are more likely to move the field forward than continued reliance on single-cell-type gain- and loss-of-function experiments alone.

## 3 Mechanisms that sustain the proliferative phase

To keep the logic of the review non-overlapping, the proliferative phase is discussed here in terms of three bounded questions: which multicellular interactions maintain the lesion, which endothelial programs support continued vascular expansion, and which macrophage survival mechanisms stabilize that niche. This separation is imperfect because the underlying biology is interconnected, but it helps prevent metabolic, inflammatory, and differentiation signals from being collapsed into a single undifferentiated growth narrative.

### 3.1 HemSC-HemEC crosstalk and the pro-angiogenic macrophage niche

One of the clearest lessons from the last decade of IH research is that proliferative growth cannot be explained solely by intrinsic endothelial abnormality. HemSCs are not just a candidate cell of origin; they actively shape the surrounding niche. In the 2015 study by Zhang and colleagues, polarized macrophages promoted HemSC proliferation and suppressed adipogenesis through Akt- and Erk1/2-dependent signaling. M2-polarized macrophages additionally promoted endothelial differentiation of HemSCs, indicating that macrophages do more than amplify existing vessels but can bias stem-cell fate toward continued vascular expansion. In the same study, adding macrophages to a murine hemangioma model increased microvessel density and delayed fat formation. These findings remain foundational because they place macrophages upstream of both growth and differentiation, thereby linking the cellular composition of the lesion to its eventual clinical trajectory (7).

The recent single-cell atlas of proliferative IH is consistent with that view. Endothelial cells appear heterogeneous and proliferative, whereas mural cells provide a major stromal compartment capable of delivering angiogenic ligands. HemSCs were not the main focus of that atlas, but the data support a model in which proliferative IH behaves as a cooperative ecosystem rather than a monoclonal endothelial mass. HemSCs likely serve as a central relay within this ecosystem, responding to stromal and immune inputs while supplying conditioned signals that influence macrophages and endothelial cells. The lesion is therefore sustained by reciprocal rather than one-way signaling (4–6).

This reciprocal model also helps explain why simplistic macrophage labeling can mislead. In the older framework, proliferative IH was often described as M2-rich and involuting IH as M1-rich. That directional trend is still useful at a broad level, but recent data suggest that macrophages in proliferative IH may occupy mixed activation states. In the ferroptosis study, HemSC-conditioned medium upregulated both CD80 and CD163, along with cytokines typically associated with both inflammatory and reparative programs. This does not negate the functional importance of M2-like signals. Instead, it suggests that the proliferative lesion may favor activated macrophage states that combine tissue-supportive survival with selective pro-angiogenic outputs. In practical terms, macrophage function may be more informative than strict binary labeling (10).

**Figure 2:**
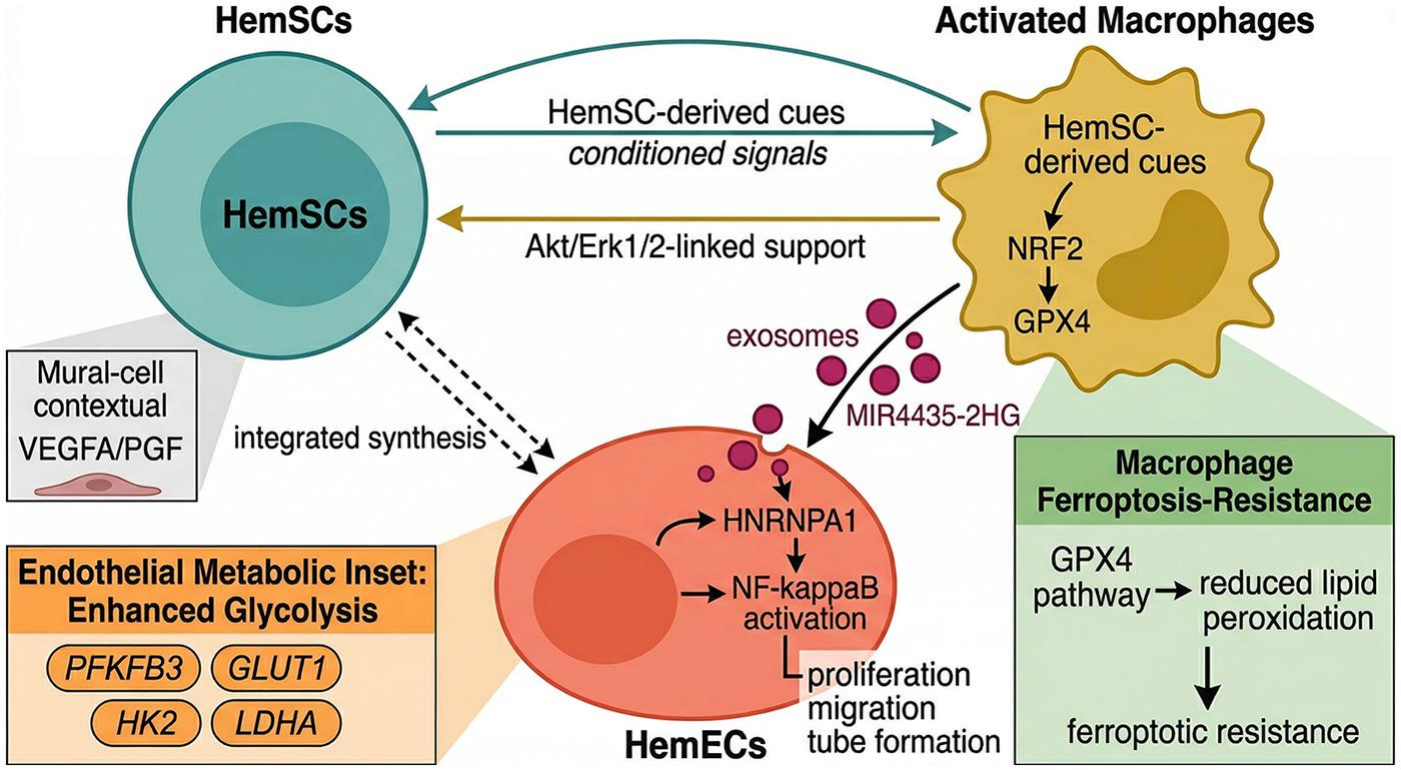
Mechanisms that sustain the proliferative niche in infantile hemangioma. The schematic shows reciprocal HemSC-endothelial-macrophage communication, including Akt/Erk1/2-linked macrophage support of HemSC behavior, M2-associated exosomal MIR4435-2HG transfer to HemECs with downstream HNRNPA1/NF-κB activation, glycolysis-associated endothelial growth programs, and NRF2-GPX4-mediated macrophage ferroptotic resistance. The figure is an interpretive schematic based on published studies, not primary experimental data.

An important implication is that the proliferative niche is self-reinforcing. HemSCs can recruit and activate macrophages, macrophages promote HemSC proliferation and endothelial differentiation, and endothelial and mural cells contribute angiogenic ligands that stabilize the growing vascular compartment. This view is supported by correlative human tissue data showing associations among macrophage markers, Ki67, VEGF, phosphorylated Akt, and phosphorylated Erk1/2. Although correlations do not prove directionality, the consistency between tissue, cell culture, and xenograft findings makes it unlikely that macrophages are incidental participants. The stronger interpretation is that proliferative IH depends on a supportive myeloid-stromal niche, and that endothelial growth is one consequence of that niche rather than its only defining feature (Figure 2).

What remains unresolved is the order of events within that niche. Current studies support macrophage participation in proliferative maintenance, but they do not yet establish whether macrophage recruitment, polarization, survival adaptation, and endothelial programming occur in a fixed sequence *in vivo* or whether multiple supportive states can coexist within the same lesion.

### 3.2 M2 macrophage exosomes and NF-κB activation in HemECs

The most direct recent evidence for macrophage-to-endothelial signaling in IH comes from the 2023 study by Li and colleagues. In that work, exosomes derived from M2-polarized macrophages were taken up by HemECs and enhanced proliferation, migration, invasion, and tube formation. Mechanistically, the exosomal cargo MIR4435-2HG bound HNRNPA1 and activated NF-κB signaling, and knockdown of MIR4435-2HG or HNRNPA1 reversed the pro-growth phenotype *in vitro* and reduced lesion growth in a nude-mouse xenograft. Human tissue staining and *in situ* localization supported the relevance of this axis to proliferative IH (12).

This study is important for several reasons. First, it offers a direct molecular route by which macrophages can influence endothelial behavior without requiring cell-cell contact. Second, it places NF-κB at a point of convergence between inflammatory signaling and endothelial growth. Third, it helps explain why macrophage effects in IH can be potent even when the absolute number of macrophages within a lesion is lower than the endothelial cell burden. Exosomal signaling is efficient, spatially permissive, and potentially amplifiable.

The strength of the evidence lies in the combined use of exosome isolation, uptake assays, RNA-seq, RNA pull-down, rescue experiments, tissue validation, and *in vivo* testing. Even so, several limitations should shape interpretation. The study used THP-1-derived M2 macrophages rather than primary infant macrophages, and it does not establish whether MIR4435-2HG is the dominant exosomal mediator in human lesions or one of several relevant cargos. It also does not define how this axis changes during involution or under propranolol exposure. Therefore, the best current conclusion is that M2-associated exosomal signaling can drive endothelial growth in IH and is mechanistically credible, not that the field has identified a universally dominant macrophage-endothelial pathway.

The translational relevance is nonetheless considerable. Exosomal pathways are attractive because they may help explain why some lesions remain biologically active despite therapies that mainly alter vascular tone or a limited set of endothelial signals. If macrophages provide durable paracrine support through RNA cargo transfer, endothelial proliferation could persist despite partial blockade of β-adrenergic or VEGF-linked pathways. At the same time, this axis has not yet been independently replicated across multiple human cohorts, and direct therapeutic targeting of exosomes or lncRNA cargo in infants remains technically and safety-wise challenging. The near-term value of this work may therefore lie more in biomarker development and biological stratification than in immediate clinical intervention. Lesions with strong M2 infiltration, high HNRNPA1 expression, or persistent NF-κB activity despite standard therapy may represent a biologically distinct subgroup worthy of closer study (Table 2).

**Table 2:** Phase-specific mechanisms linked to proliferation and involution in infantile hemangioma.

Biological domain	Proliferative-phase pattern	Involuting/remodeling pattern	Evidence status	Representative references
Macrophage compartment	Increased macrophage burden; macrophage support of HemSC proliferation and endothelial differentiation; M2-associated exosomal growth signaling; GPX4-high activated macrophages	Relative increase in M1-linked inflammatory remodeling signals; reduced GPX4 and higher 4-HNE in lesion-associated macrophages	Phase association is supported; exact *in vivo* sequence is a working model	(7, 8, 10, 12)
Endothelial state	Dense vascular endothelial compartment with strong angiogenic behavior; partial mesenchymal marker induction can still coexist with migration and tube formation	Loss of stable endothelial identity, inflammatory EndMT signatures, and later adipogenic competence	Context dependent; not all EndMT-like change is regressive	(8, 9, 20)
HemSC, mural, and perivascular fate bias	HemSC endothelial differentiation favored; adipogenesis restrained; mural and perivascular cells retain proangiogenic support capacity	Adipogenic differentiation and fibrofatty remodeling become more apparent as vascular programs weaken	Mixed evidence from tissue and patient-derived models; temporal ordering partly inferred	(4–7, 11)
Dominant metabolic features	Enhanced glycolytic dependence in HemECs; increased glycolysis-associated molecule expression; altered amino acid handling supports biomass and redox balance	Late-stage metabolic remodeling is less well defined; lipid handling and oxidative injury become more visible than clear late glycolytic signatures	Proliferative glycolysis is established; involuting metabolic transition remains incompletely mapped	(10, 13, 14, 20)
Oxidative stress and ferroptosis-related features	Low 4-HNE and macrophage-enriched GPX4/NRF2 activity support macrophage persistence in proliferative lesions	Higher 4-HNE with lower GPX4 is associated with involution	Human association is supported; lesion-wide causal sequence remains model-supported	(10)
Integrated lesion-state model	Cooperative HemSC-HemEC-mural-macrophage niche supports vascular persistence and growth	Niche destabilization, inflammatory remodeling, and adipogenic conversion contribute to regression	Author synthesis based on cross-sectional tissue plus mechanistic studies	(4, 7, 8, 10–12)

### 3.3 Metabolic rewiring of HemECs: glycolysis, amino acid use, and growth signaling

The metabolic literature in IH is uneven in quality, and the relevant evidence spans different tiers. The best-supported signal is enhanced glycolytic dependence in proliferative endothelial cells. In 2023, Yang and colleagues reported higher PFKFB3 expression in proliferative IH tissue and HemECs than in involuting tissue and HUVEC controls. Pharmacologic inhibition with PFK15 reduced glycolytic flux, suppressed angiogenic behavior and migration, induced apoptosis, and decreased xenograft growth. The same study reported synergy between PFK15 and propranolol. The human-tissue component supports disease association, whereas the HemEC and xenograft experiments support mechanistic plausibility rather than direct clinical efficacy. Earlier work showing increased expression of glycolysis-associated molecules in IH supports the view that PFKFB3 is part of a broader endothelial metabolic program (13, 14).

The second consistent theme is altered nutrient handling beyond glucose. Integrated untargeted and targeted metabolomics identified broad metabolite differences between HemECs and HUVECs, with enrichment of amino acid pathways rather than a glucose-only signature. Differentially abundant metabolites included glutamine, arginine, asparagine, and serine, and enriched pathways included alanine/aspartate/glutamate metabolism, arginine biosynthesis, arginine/proline metabolism, and glycine/serine/threonine metabolism. The most defensible interpretation is not that any one amino acid is uniquely essential, but that proliferative HemECs adopt a broader anabolic program that supports biomass accumulation, redox balance, and nucleotide synthesis. This matters because glycolysis in endothelial biology often serves biosynthetic as well as energetic functions.

Where the evidence becomes more speculative is at the level of upstream regulatory nodes. Several recent studies propose specific drivers of glycolytic activation. MCM3AP-AS1 was reported to promote IH progression by modulating the miR-138-5p/HIF-1α axis. OTUB1 promoted angiogenesis and glycolysis through catalytic-independent deubiquitination of TGFBI. IL13RA2 enhanced proliferation, migration, invasion, and glycolysis while activating Wnt/β-catenin signaling. ALKBH5 promoted FOXF1 expression in an m6A-YTHDF2-dependent manner, leading to HK2 activation, and HECW2 was shown to enhance glycolysis through ALKBH5-mediated stabilization of LDHA expression. These studies converge on a recognizable metabolic program centered on GLUT1, HK2, LDHA, PDK1, and glycolytic flux. However, most are single-axis studies from limited model systems, often using overexpression or knockdown in cultured cells plus xenografts. They should therefore be interpreted as plausible mechanistic candidates rather than settled field consensus (15–19).

The question is how to extract value from this crowded mechanistic landscape without inflating certainty. A reasonable synthesis is that proliferative IH displays a reproducible glycolytic phenotype, whereas the exact upstream architecture remains incompletely ranked. HIF-1α, Wnt/β-catenin, PI3K/AKT signaling, m6A regulation, and ubiquitin-related control may all contribute, but the field does not yet know whether these pathways operate in parallel, in sequence, or in lesion-specific subsets. It is also unclear which of these axes remain active *in vivo* under propranolol treatment. The most defensible conclusion is therefore that enhanced glycolysis is likely a disease-relevant feature of proliferative HemECs, while many individual upstream regulators still require external validation across independent cohorts and platforms.

Some of the apparent disagreement within the metabolic literature likely reflects methodology rather than true biological contradiction. Expression-oriented studies tend to highlight upstream regulatory candidates, whereas flux assays and inhibitor studies identify nodes that are functionally important for short-term cell survival or angiogenic behavior. Xenograft experiments then add a third readout, namely lesion-forming capacity. These are related but not interchangeable endpoints. A pathway that alters the extracellular acidification rate in vitro is not automatically the same as a pathway that determines lesion persistence *in vivo*, and not every regulator identified in one model should be treated as a universal driver of human disease.

This caution does not lessen the translational significance of the metabolic data. In fact, it clarifies where the therapeutic signal is strongest. At present, the best-supported intervention point is not any one newly described upstream gene, but the broader glycolytic dependency itself and the linked PI3K/AKT/mTOR program. Propranolol appears to influence this network indirectly through β-adrenergic signaling, HIF-1α reduction, endothelial apoptosis, and altered HemSC differentiation. Earlier studies showed that propranolol promotes apoptosis in HemECs and accelerates adipogenic differentiation in HemSCs, although the adipogenic program it induces may be incomplete or dysregulated rather than equivalent to normal adipose maturation. The practical implication is that metabolic control is likely one contributor to treatment response and heterogeneity in IH, not a standalone explanation for all propranolol sensitivity or resistance (2, 21, 22, 25).

### 3.4 Ferroptotic resistance as a macrophage survival mechanism

The 2025 study by Liu and colleagues adds a distinct but highly relevant layer to this picture by shifting attention from endothelial metabolism to macrophage survival. The core observation was phase specific: involuting IH showed higher 4-hydroxynonenal staining, consistent with greater lipid peroxidation, whereas proliferative IH showed higher GPX4 expression, particularly within CD68-positive macrophages. *In vitro*, HemSC-conditioned medium induced GPX4 expression in macrophages through NRF2 nuclear translocation, reduced lipid peroxidation, and improved viability under ferroptotic stress. *In vivo*, GPX4 knockdown or the ferroptosis inducer RSL3 reduced lesion growth, vascular density, and macrophage infiltration in a nude-mouse model (10).

This study is notable because it adds more than another cell-death pathway to IH. It proposes that proliferative lesions preserve their supportive immune compartment by protecting macrophages from ferroptotic loss. That is a meaningful conceptual advance. If macrophages are required to sustain HemSC proliferation, endothelial differentiation, or exosomal signaling, then protecting macrophage survival becomes a lesion-maintenance strategy in its own right. In this sense, macrophage ferroptotic resistance may be upstream of multiple downstream growth pathways.

The data are also interesting because they complicate canonical M1/M2 interpretation. HemSC-conditioned medium increased both CD80 and CD163 expression, along with cytokines associated with inflammatory and reparative activation states. Thus, the survival advantage conferred by GPX4 cannot be reduced to a pure M2 program. A more defensible interpretation is that proliferative IH preserves activated macrophage populations with mixed phenotypic features, and GPX4 allows those cells to persist within an oxidatively stressful microenvironment. Whether those macrophages then exert predominantly M2-like or M1-like effects may depend on lesion stage and local cues. In proliferative disease, the functional consequences appear growth supportive.

Important limitations remain. The macrophage experiments relied heavily on THP-1-derived cells and conditioned-medium systems, and direct longitudinal evidence in human infants is absent. It is also not known whether propranolol modulates macrophage GPX4 or whether the GPX4-high macrophages identified in human lesions correspond to specific ontogenically defined subsets. Nevertheless, among recent immune studies in IH, this is one of the most clinically provocative because it identifies a potentially targetable survival mechanism with *in vivo* consequences. The main translational caveat is safety: systemic induction of ferroptosis in infants is not an acceptable near-term strategy without lesion-selective delivery and much better toxicity modeling. For now, macrophage ferroptotic resistance should be viewed as a promising mechanistic vulnerability and a candidate biomarker axis, not as an immediately actionable treatment target (Figure 2).

The established component of this literature is therefore phase-linked enrichment of GPX4 within the macrophage compartment and model-based growth suppression when that pathway is disrupted. The broader claim that macrophage ferroptotic resistance is a central organizing principle of proliferative IH remains a plausible working model that still requires validation in longitudinal and spatially resolved human studies.

## 4 Mechanisms linked to involution and fibrofatty remodeling

Processes linked to involution are considered here as active remodeling events rather than passive disappearance of proliferative vessels. This separation is useful because some pathways discussed below represent durable exit from the vascular program, whereas others may instead reflect transient or partial state change that still supports angiogenic behavior. Keeping those possibilities separate reduces the risk of reading all late-stage marker changes as evidence of the same biological endpoint.

### 4.1 Endothelial plasticity and EndMT during regression

The shift from proliferative IH to involution has long been described histologically, but the mechanistic route from vascular lesion to fibrofatty residuum remained obscure until studies began to examine endothelial plasticity more directly. Wu and colleagues provided a major advance by showing that M1-polarized macrophages induce EndMT and promote regression-associated changes in IH. *In vitro*, M1-conditioned medium, TNF-α, and to a lesser extent IL-1β and interferon-*γ* reduced endothelial markers and increased mesenchymal markers in endothelial cells. In patient samples, involuting lesions showed perivascular M1 infiltration together with endothelial expression of Snail/Slug. Most notably, M1-treated HemECs acquired the capacity to undergo adipogenic differentiation under appropriate induction conditions, whereas untreated HemECs did not. This work gave the field a biologically plausible bridge from inflammation to adipose replacement (8).

The clinical appeal of this model is obvious. It explains involution not as passive endothelial attrition, but as an active reprogramming event in which inflammatory cues loosen endothelial identity and permit lineage redirection. It also aligns with the long-recognized observation that involuted lesions leave behind fibrofatty tissue rather than simply disappearing. However, newer data show that EndMT in IH cannot be interpreted as uniformly beneficial or uniformly regressive.

The clearest example comes from the 2025 TGF-β1 study by Gong and colleagues. In primary proliferative HemECs, TGF-β1 induced loss of endothelial markers and gain of mesenchymal markers, yet this shift was accompanied by increased migration, invasion, tube formation, and *in vivo* vessel formation. The same study linked TGF-β1-induced changes to altered lipid metabolism, reduced CPT1A expression, and increased lipid accumulation. On first reading, these findings appear to contradict the regression model proposed by Wu and colleagues in 2017. In fact, the discrepancy is likely explained by differences in upstream signal, starting cell state, exposure duration, and endpoint definition. The M1 study emphasized inflammatory cytokines and later adipogenic competence, whereas the TGF-β1 study emphasized short-term motility, angiogenesis, and lipid-handling changes in proliferative HemECs. EndMT in IH is therefore better interpreted as a spectrum of endothelial-state transitions than as a single regressive switch. Inflammatory EndMT driven by TNF-α-rich M1 signals may facilitate durable loss of endothelial identity and later adipogenic competence, whereas TGF-β1-driven partial EndMT in proliferative HemECs may increase motility and matrix remodeling while preserving or even enhancing angiogenic potential (8, 20).

**Figure 3:**
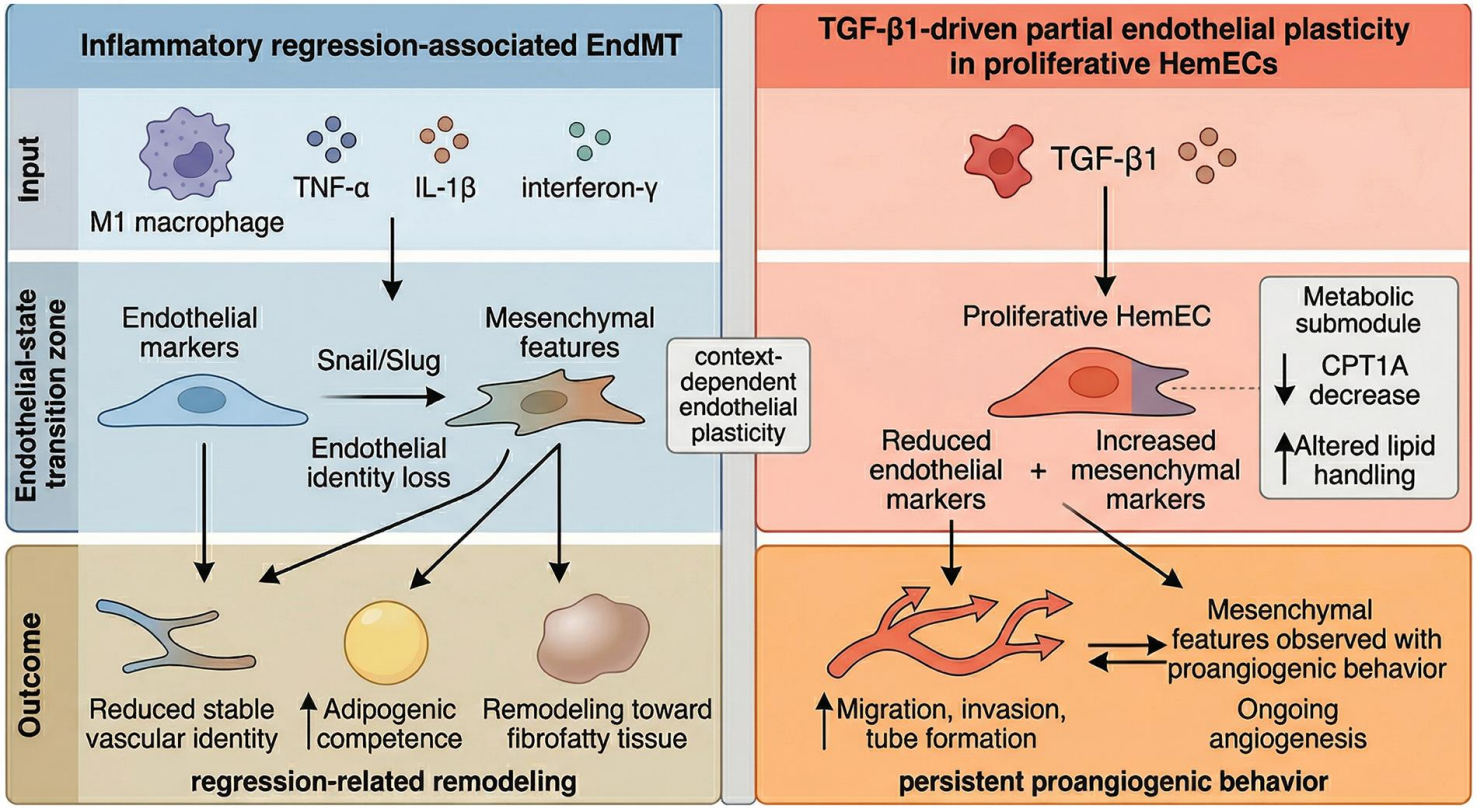
Context-dependent endothelial plasticity during infantile hemangioma remodeling. The left panel summarizes inflammatory, regression-associated EndMT driven by M1 macrophage-related cytokines, with loss of endothelial identity, mesenchymal marker acquisition, adipogenic competence, and fibrofatty remodeling. The right panel summarizes TGF-β1-driven partial endothelial plasticity in proliferative HemECs, where mesenchymal marker induction can coexist with migration, invasion, tube formation, altered lipid handling, and ongoing angiogenic behavior. The figure is a schematic representation of published mechanisms and does not present primary experimental data.

Clinically, this distinction is important. If EndMT markers are measured without regard to phase, upstream driver, or functional endpoint, they can be misclassified as either pro-regression or pro-proliferation. Interpretation therefore requires careful attention to biological context. The field currently has good evidence that endothelial plasticity contributes to IH biology, and reasonable evidence that inflammatory EndMT can support regression. It does not have evidence that all mesenchymal marker acquisition in IH is regressive. In practice, EndMT should be treated as a context-dependent process whose consequences depend on the starting endothelial state, the cytokine environment, and the duration of signaling exposure (Figure 3).

### 4.2 Adipogenic differentiation and loss of the proliferative vascular program

The final clinical outcome of involution is fibrofatty tissue, so any complete model of IH regression must explain adipogenesis as well as vascular loss. HemSC biology remains central here. HemSCs can differentiate into endothelial cells, pericytes, and adipocytes, and several studies suggest that the balance among these fates helps determine lesion behavior. The 2024 KLF2 study is particularly informative because it links a defined transcription factor to this balance. KLF2 was highly expressed in endothelial cells from proliferative IH and decreased in adipocytes from involuting lesions. In patient-derived HemSCs and implantation models, KLF2 promoted proliferation and favored endothelial differentiation while restraining adipogenesis. Knockdown induced a proadipogenic transcriptome, impaired vessel formation, and accelerated adipocyte differentiation. These findings support the idea that involution requires active release from a vascular-maintaining transcriptional program (11).

Propranolol intersects with this biology in a clinically relevant way. Earlier studies showed that propranolol increases apoptosis in HemECs and accelerates adipogenic differentiation of HemSCs. However, later work suggested that the adipogenesis induced by propranolol may be dysregulated rather than equivalent to normal terminal adipocyte maturation, with evidence of altered expression of RXR and C/EBP family members and increased cell death within differentiating HemSCs. This nuance matters because it implies that drug-induced lesion regression may not reproduce spontaneous involution. Instead, propranolol may force exit from the proliferative vascular program through a combination of endothelial injury, altered β-adrenergic signaling, metabolic stress, and partial adipogenic redirection (21, 22).

The KLF2 and propranolol data therefore suggest that involution is best understood as loss of vascular identity plus imperfect acquisition of adipose fate, rather than a clean binary switch from vessel to fat. This interpretation accommodates the clinical reality that involuted lesions often leave variable residual tissue rather than a uniform adipose replacement. It also helps explain why therapies that shrink vascular volume do not necessarily eliminate contour deformity. For translational purposes, the relevant question may not be how to induce adipogenesis *per se*, but how to guide a controlled exit from the proliferative state without worsening fibrofatty sequelae.

### 4.3 Interpreting the shift from macrophage support to macrophage restraint

The macrophage literature in IH often reads as though the field has already settled on a simple temporal switch: M2 macrophages sustain proliferation, and M1 macrophages drive regression. The broad outline is probably directionally correct, but the actual biology appears more layered. The strongest evidence for proliferative support comes from the 2015 macrophage-HemSC study, the 2023 M2 exosome study, and the 2025 macrophage ferroptosis study. Together, these show that macrophages can promote HemSC proliferation, endothelial differentiation, exosomal growth signaling, and survival within the lesion. The strongest evidence for regression-linked macrophage activity comes from the 2017 M1-EndMT study, which tied M1 cytokines to endothelial plasticity and adipogenic competence in involuting tissue.

What remains missing is longitudinal proof that the same lesion passes through a clearly ordered macrophage-state sequence *in vivo*. The current evidence is cross-sectional, model-based, or both. Moreover, the ferroptosis study showed mixed marker induction rather than clean canonical polarization, and the lesion microenvironment almost certainly contains macrophages that do not fit textbook M1/M2 boundaries. The most cautious synthesis is therefore a working model rather than a settled conclusion: proliferative IH likely depends on activated macrophage populations that are protected from oxidative death and whose net functional output favors endothelial growth and maintenance of a vascular niche. As lesions involute, oxidative stress rises, GPX4-mediated protection falls, and inflammatory programs capable of destabilizing endothelial identity become more apparent. The balance shifts from macrophage-supported vascular persistence to macrophage-linked vascular remodeling and regression.

This working model is attractive because it integrates otherwise disconnected observations: higher macrophage burden in proliferative lesions, M2-associated exosomal signaling, GPX4-high macrophages in proliferative tissue, increased 4-HNE in involution, and M1-associated EndMT in regressing lesions (7, 8, 10, 12). But it also identifies a major evidence gap. No current study tracks macrophage states, endothelial states, and metabolic changes longitudinally in the same human lesions, and no spatial study has yet shown whether GPX4-high macrophages, EndMT-prone endothelial cells, and adipogenic regions occupy linked microanatomic niches. Until such data exist, the field should resist claiming that macrophage switching alone explains involution. It is more likely that macrophage behavior is one part of a broader niche collapse that includes metabolic reprogramming, stromal change, and loss of endothelial fate stability.

## 5 Translational implications

### 5.1 Biomarkers for phase assignment, risk stratification, and treatment response

The immediate translational challenge in IH is that clinically important decisions are often made without tissue, whereas most mechanistic discoveries still come from tissue-based studies. Any proposed biomarker strategy must therefore distinguish between what is biologically informative and what is clinically deployable. GLUT1 remains diagnostically useful in tissue, but it is not a dynamic biomarker of phase transition. Ki67, VEGFA, and endothelial density reflect proliferative burden, but again they require lesion material. The emerging macrophage-metabolic markers are promising precisely because they may add phase information rather than simple diagnosis. GPX4-positive CD68-positive macrophages and low 4-HNE are associated with proliferative lesions, whereas higher 4-HNE and lower GPX4 are associated with involution. Likewise, KLF2-rich endothelial programs align with vascular maintenance, whereas Snail/Slug and PPAR-*γ*-associated states suggest transition toward remodeling (1, 8, 10, 11).

However, it would be premature to advocate routine tissue biomarker panels for all patients. Most IHs are diagnosed clinically, and biopsy is neither necessary nor desirable in standard cases. The more realistic translational use of tissue markers is in selected high-risk, atypical, refractory, or surgically treated lesions, where correlative studies can inform biology and future stratification. For noninvasive biomarker development, the field should focus on analytes or imaging features that can plausibly track phase and treatment response. Earlier serum work showed that VEGF, bFGF, MMP-2, and MCP-1 decline during propranolol treatment, suggesting that circulating factors may have value, although their specificity is limited (23, 24). The next generation of biomarker work should test whether circulating cytokines, extracellular vesicle cargo, or targeted metabolite panels correlate with ultrasound flow, growth rate, and treatment response in prospectively followed infants.

A practical biomarker framework would therefore be layered. Tissue biomarkers should define biological states in discovery cohorts; blood-based and imaging biomarkers should then be mapped onto those states in clinically accessible cohorts. For example, a proliferative high-support lesion might be defined in tissue by strong endothelial proliferation, mural VEGFA/PGF signaling, GPX4-high macrophages, and a glycolytic signature, whereas a remodeling lesion might show higher lipid peroxidation, inflammatory macrophage markers, reduced vascular transcriptional programs, and adipogenic features. Only after such state definitions are stable should the field attempt to derive reduced biomarker panels suitable for routine care (Table 3).

**Table 3:** Translational targets, candidate biomarkers, and key caveats.

Translational axis	Proposed clinical use	Strongest supporting evidence	Current limitation or risk	Evidence status	Representative references
Early risk stratification and referral	Identify lesions needing prompt specialty evaluation and early treatment	Clinical guideline and established high-risk lesion framework	Does not by itself define lesion biology or predict drug response heterogeneity	Established clinical practice	(1)
Propranolol-centered β-blockade	First-line systemic treatment for problematic proliferative IH	Randomized propranolol trial; class-level support from nadolol noninferiority; large clinical experience	Rebound and incomplete response still occur; mechanism is pleiotropic rather than single-pathway	Established clinical practice	(2, 3, 27)
Tissue macrophage oxidative-stress panel (CD68, GPX4, 4-HNE)	Phase stratification in selected tissue-based or resected-lesion contexts	Human phase comparison plus model-supported GPX4 perturbation	Biopsy is not routine; no validated noninvasive surrogate	Candidate tissue biomarker	(10)
PFKFB3 and endothelial glycolysis	Add-on strategy for refractory proliferative lesions; mechanism-aware escalation	Tissue expression, flux suppression, apoptosis, xenograft inhibition, and propranolol synergy	Entirely preclinical in IH; systemic toxicity and pediatric dosing unresolved	Preclinical therapeutic candidate	(13, 14)
PI3K/AKT/mTOR pathway	Potential escalation target where vascular maintenance programs remain active	Macrophage-HemSC signaling work and exploratory drug-screen support	No IH-specific clinical trial evidence; screening endpoint is not the same as clinical lesion control	Preclinical therapeutic candidate	(7, 29)
M2 exosomal MIR4435-2HG/HNRNPA1/NF-κB axis	Biological subgrouping and future lesion-targeted intervention	Direct exosome uptake, rescue experiments, tissue validation, xenograft growth effects	Currently supported mainly by one mechanistic study; delivery strategy unclear	Early mechanistic candidate	(12)
Macrophage GPX4/NRF2 ferroptotic resistance	Future niche-destabilizing strategy in highly macrophage-dependent lesions	Human phase data plus *in vitro* and *in vivo* perturbation	Infant safety, lesion selectivity, and macrophage subset specificity are unresolved	Preclinical therapeutic candidate	(10)
KLF2-linked fate regulation	Marker of vascular-state persistence and possible future differentiation-based intervention	Human tissue, patient-derived HemSC assays, and implantation models	Not clinically actionable at present; requires validation against treatment response and phase	Emerging mechanistic marker	(11)
Noninvasive state tracking with imaging and circulating markers	Monitor response, rebound risk, and phase transition without routine biopsy	Clinical need is clear; serum cytokine shifts and imaging metrics are biologically plausible	Specificity remains low and no integrated biomarker panel is validated	Author synthesis with limited early support	(1, 3, 23, 24)

### 5.2 Therapeutic vulnerabilities across immune, metabolic, and cell-state axes

At present, propranolol is the only intervention discussed in this review with established routine clinical utility in IH. All other targets below should be read as translational candidates whose level of support depends heavily on whether the evidence comes from human tissue, patient-derived cells, or lower-fidelity model systems.

Therapeutic translation in IH should begin from what is already clinically effective. Propranolol remains the standard against which new strategies must be judged, and its biology is broader than simple vasoconstriction. Experimental data indicate that propranolol can reduce HIF-1α signaling, suppress endothelial survival pathways, alter cAMP and MAPK signaling in HemSCs, promote endothelial apoptosis, and push stem cells away from a proliferative vascular program (2, 21, 22, 25). These diverse actions may help explain why a single drug can be effective in a biologically complex lesion. They also imply that treatment resistance may emerge when alternative niche-supporting pathways remain active. This concern has clinical support. Observational outcome data and newer analyses of treatment timing both indicate that a subset of lesions continue to grow or respond inadequately despite standard β-blockade (26, 28). At the same time, the randomized noninferiority trial comparing nadolol with propranolol indicates that β-blockade remains the clinically validated therapeutic class even as its downstream biology is refined (27).

Among metabolic targets, PFKFB3 is currently the most actionable preclinical candidate because its role is supported by tissue expression, cell-based metabolic assays, apoptosis studies, synergy with propranolol, and *in vivo* suppression of lesion growth (13, 14). PI3K/AKT/mTOR signaling is another rational axis, particularly because it sits downstream of both growth-factor and metabolic programs. The 2025 immortalized HemEC (iHemEC) drug-screening study identified everolimus and sunitinib as more potent than propranolol or rapamycin in that model, but these findings should be weighed differently. They derive from a screening platform rather than patient-outcome data and should therefore be treated as prioritization signals rather than treatment recommendations (29). Everolimus aligns with a recognizable pathway and has precedent in pediatric vascular anomaly care outside IH, whereas sunitinib induced chromosome instability and DNA damage *in vitro*, which makes it biologically interesting but less attractive as an immediate translational candidate for infants. The proper inference is not that these agents are ready for widespread clinical use in IH, but that phase-matched pathway inhibition deserves formal study.

That distinction between screening efficacy and clinical usefulness is especially important in IH. Cell-viability or tube-formation assays test whether an intervention disrupts a model phenotype, whereas clinicians care about safer and earlier control of a lesion's growth trajectory, prevention of functional compromise, and reduction of rebound or residual deformity. A review that does not distinguish these endpoints will tend to overvalue preclinical potency. For this reason, the translational weight given here to everolimus, sunitinib, or any future pathway-directed agent is intentionally lower than the weight given to propranolol, even when laboratory models show stronger immediate inhibitory effects.

Immune targets are equally compelling but more complex. Inhibiting M2-associated exosomal signaling, reprogramming macrophages, or weakening NRF2-GPX4-mediated macrophage survival could all, in principle, destabilize the proliferative niche (10, 12). Yet systemic immune manipulation or ferroptosis induction in infants raises obvious safety concerns. For that reason, the most realistic near-term role of these pathways may be to define which lesions are particularly dependent on macrophage support, thereby guiding combination strategies or future lesion-targeted delivery systems. The field should also be careful not to assume that pushing macrophages toward an M1-like state is always beneficial, because uncontrolled inflammatory remodeling could worsen ulceration or local tissue injury.

Cell-state targets offer a third translational lane. KLF2-related fate regulation, TGF-β/CPT1A-linked endothelial plasticity, and adipogenic programs suggest that IH may be manipulated through endothelial-cell loss and through redirection of differentiation (11, 20–22). This is conceptually attractive because the clinical endpoint in IH is not eradication of a malignant clone but durable transition out of a harmful growth state. However, the current evidence base is not yet sufficient to justify direct manipulation of these pathways in infants. The safer translational use is to treat them as mechanistic readouts that can help explain why some therapies succeed or fail.

### 5.3 Implications for combination therapy and early-phase trial design

If IH is maintained by a cooperative niche, combination therapy should be designed to interrupt more than one supportive process at a time. The most rational current combinations are those that pair standard care with a second agent aimed at a well-supported dependency. On present evidence, propranolol plus a glycolytic inhibitor or propranolol plus an mTOR-pathway inhibitor is more defensible as a translational hypothesis than empiric pairing with broad cytotoxic or immune-disruptive agents. The PFK15-propranolol synergy study supports this logic preclinically, and the everolimus screening data suggest that mTOR inhibition deserves closer evaluation in propranolol-refractory disease, but neither approach can yet be regarded as clinically validated in IH (13, 29).

For metabolic inhibitors, the clinically realistic path is an add-on strategy for carefully selected proliferative IH rather than replacement of established β-blockade. PFKFB3 inhibition has the strongest IH-specific preclinical signal, but no PFKFB3 inhibitor has been validated for infants with IH. Any pediatric use would require pharmacokinetic data, off-target metabolic safety assessment, and clear stopping rules. A cautious development sequence would first test whether PFKFB3 expression or glycolytic signatures identify poor early responders to propranolol, then evaluate short-course add-on therapy in refractory high-risk lesions using imaging-based growth control and safety endpoints. Similar caution applies to mTOR-pathway inhibition: pathway logic and drug-screening data justify further study, but routine clinical use should wait for IH-specific safety and efficacy data.

Trial design should be phase aware. The ideal early-phase cohort would consist of infants with high-risk proliferative IH who either have an inadequate early response to propranolol or are predicted to be poor responders on the basis of lesion growth kinetics and imaging. Endpoints should go beyond photographic change. Serial ultrasound flow metrics, lesion thickness, ulceration status, and clinically meaningful functional measures should be paired with blood biomarkers and, where clinically indicated, tissue correlates. Because routine biopsy is not appropriate, correlative tissue programs should be embedded opportunistically in surgical debulking or diagnostic procedures rather than mandated for all participants.

The most important translational principle is that mechanism should guide escalation. Lesions showing strong evidence of persistent proliferative vascular biology may be the most plausible candidates for metabolic add-on strategies, whereas lesions already entering remodeling states may not benefit from the same intervention and could instead be monitored or managed surgically for residuum. This implies that future trials should incorporate biological stratification from the beginning rather than treat IH as a single therapeutic category. A phase-stratified approach would be more aligned with disease biology and more likely to produce interpretable results than broad inclusion of any lesion judged clinically severe. It may also help explain rebound and incomplete response, which are unlikely to arise from a single shared mechanism across all lesions.

## 6 Current challenges and future perspectives

The main obstacle in IH biology is no longer absence of candidate mechanisms. It is the lack of integration across phase, cell type, and evidence level. The field has identified plausible roles for macrophage crosstalk, glycolysis, m6A regulation, ubiquitin-associated signaling, endothelial plasticity, and stem-cell fate control. What remains uncertain is how these processes are organized in human lesions over time. Most studies still compare one proliferative group with one involuting group, often with modest sample numbers and limited clinical annotation. That design can identify associations, but it cannot reconstruct the trajectory by which a lesion changes state.

The most informative next human study over the coming three to five years would therefore be a prospective, multicenter, phase-resolved cohort with tightly phenotyped untreated proliferative lesions, early propranolol responders, poor responders or rebound cases, and involuting lesions. Imaging, serum, and clinically indicated tissue should be collected in parallel. The key aim would be to map coordinated lesion states rather than generate another list of differentially expressed genes. Specifically, such a cohort should test whether GPX4-high macrophages, VEGFA/PGF-rich mural compartments, glycolytic endothelial programs, and KLF2-high vascular states co-occur in fast-growing lesions, and whether increasing 4-HNE, inflammatory macrophage programs, Snail/Slug expression, and adipogenic signatures define biologically distinct remodeling states.

Spatial validation is the most pressing technical need. Bulk tissue and even single-cell data cannot show whether the relevant macrophage, endothelial, and stromal states are actually interacting *in situ*. Spatial transcriptomics or multiplex imaging should be focused on a small number of concrete questions rather than used as a generic discovery exercise. The first priority should be to define where GPX4-positive macrophages sit relative to endothelial sprouts, HemSC-rich regions, and mural-cell ligand sources. The second should be to distinguish inflammatory EndMT associated with involution from TGF-β-linked partial EndMT associated with continued angiogenic behavior. The third should be to identify where adipogenic markers emerge relative to collapsing vascular regions. These are tractable questions with direct implications for therapy.

Model improvement is equally necessary. THP-1-derived macrophages, HUVEC comparators, and short-term monocultures have been useful, but they distort the lesion context. The next generation of mechanistic studies should use patient-derived multicellular systems that include HemSCs, HemECs, mural cells, and primary macrophages whenever feasible. Organotypic explants and improved co-culture models will be more informative than additional single-axis overexpression papers. The new immortalized HemEC model may be valuable for drug screening, but it must be benchmarked carefully against primary cells and human tissue before its therapeutic implications are weighted heavily (4, 29).

Several field-level overinterpretations should be actively avoided. The first is treating M1 and M2 labels as if they fully describe macrophage behavior in IH. Available data already suggest mixed activation states. The second is assuming that all EndMT is regressive. Current evidence clearly indicates context-dependent outcomes. The third is presenting newly reported glycolytic regulators as though each were a validated master switch. Many of these pathways are promising, but most are supported by single-study evidence and need independent replication. The fourth is assuming that drug potency in a cell line or xenograft predicts acceptability in infants. For example, the *in vitro* activity of sunitinib is interesting, but the associated DNA-damage signature should increase rather than decrease translational caution.

From a therapeutic standpoint, the most actionable mechanistic vulnerability at present is likely phase-specific metabolic dependence, particularly where it intersects with established pediatric pharmacology. PFKFB3 and PI3K/AKT/mTOR pathways are more ready for disciplined translational follow-up than broad ferroptosis induction or unspecific macrophage manipulation. A rational early-phase strategy would therefore test add-on therapy in propranolol-refractory, high-risk proliferative IH using strong correlative endpoints, not immediately pursue systemic immune reprogramming in unselected infants. By contrast, macrophage GPX4 and exosomal lncRNA pathways should remain high-priority preclinical targets until lesion-selective delivery and safety are better defined.

More broadly, the field is moving toward a state-based understanding of IH. Instead of asking which single gene causes the disease, the more useful question is which combinations of endothelial, stromal, immune, and metabolic states maintain proliferation or permit involution. That shift should improve both mechanistic clarity and clinical decision making. It will also make the field less vulnerable to the recurrent problem of overinterpreting elegant but isolated pathway studies.

## 7 Conclusion

The unresolved problem in infantile hemangioma is more than excessive angiogenesis. It is the persistence of a phase-specific supportive niche that allows some lesions to remain highly proliferative before eventually transitioning toward involution and fibrofatty remodeling. Current evidence supports a framework in which macrophage crosstalk, endothelial metabolic rewiring, and cell-state plasticity jointly determine that transition. Proliferative IH appears to depend on HemSC-HemEC-macrophage cooperation, enhanced glycolysis, and macrophage ferroptotic resistance, whereas involution is associated with increased lipid peroxidation, loss of vascular fate stability, inflammatory remodeling, and adipogenic differentiation. At the same time, several widely used shorthand interpretations remain too simplistic: macrophage states are not fully captured by an M1/M2 binary, and EndMT is not uniformly regressive.

What this review clarifies is the need to interpret IH as a dynamic multicellular process rather than a single-pathway vascular anomaly. What remains uncertain is the temporal ordering of these events in human lesions and the extent to which they are altered by standard therapy. The near-future trajectory of the field should therefore center on phase-resolved human cohorts, spatially anchored validation of immune and endothelial states, and carefully stratified translational studies built around the most reproducible metabolic and signaling dependencies. That path is more likely to yield clinically useful biomarkers and rational combination therapies than continued accumulation of disconnected mechanism papers.
